# Informal learning and wellbeing outcomes of gameplay and their associations with gameplay motivation

**DOI:** 10.3389/fpsyg.2023.1176773

**Published:** 2023-05-30

**Authors:** Jukka Vahlo, Tanja Välisalo, Kai Tuuri

**Affiliations:** ^1^Centre for Collaborative Research, School of Economics, University of Turku, Turku, Finland; ^2^Department of Music, Art and Culture Studies, University of Jyväskylä, Jyväskylä, Finland; ^3^Faculty of Education and Psychology, University of Jyväskylä, Jyväskylä, Finland

**Keywords:** informal learning, non-educational games, gameplay motivation, wellbeing outcomes, self-determination

## Abstract

Educational functions of digital games are often seen only in the light of the serious and purposeful activities that aim for learning outcomes, in contrast with non-educational games that are designed for entertainment. The focus of this paper is in studying players’ learning outcomes from playing non-educational games, and how these relate to wellbeing outcomes of playing, and gaming motivation. The data for this study was collected via a survey (*N* = 1,202) in the United Kingdom and the United States. The survey respondents answered the question regarding what players perceive they have learnt by playing digital games. A generic data-driven qualitative content analysis of the responses to this question yielded 11 categories representing different types of game-based learning outcomes. A consequent cluster analysis suggested three groups of informal game-based learning, which differed in their emphasis on (1) learning persistence, (2) learning practices and community, and (3) learning to perform. Our analyses indicated substantial connections between the learning outcomes and gameplay motives and gameplay activity preferences. Such connections point out how gameplay activity has an inherently close relationship with learning. Moreover, the results yielded significant association between learning outcomes, wellbeing measures, and eudaimonic motives to play digital games. These results indicate that playing games because gaming is aligned with players’ core values and need for self-realization are clear precedents for both wellbeing and learning outcomes.

## 1. Introduction

In comparison to purposeful activities of individuals and communities, in general, play does not seem to associate with clear goals. Play appears to epitomize humans’ desire to act on a purely voluntary basis—often just for fun. However, this is not to say that play would not have any benefits. Play has been associated with spontaneous learning in activities that may seem aimless (e.g., Piaget, 1952; Lieberman, 1977). In contrast to play, the educational view on learning often emphasizes a formal curriculum, in which certain goals are to be reached. Likewise, the prevailing conceptualization of digital games tends to differentiate the domains of entertainment and serious gaming. Thus, educational functions of digital games are often seen only in the light of the serious and purposeful activities that aim for learning outcomes, in contrast with non-educational games that are designed for entertainment. The focus of the paper is in building a bottom-up understanding of what players perceive they have learnt by playing digital games, without any pre-condition that these games should or would be educational in the first place. Secondly, we are interested in how these informal learning outcomes might be associated with wellbeing and with motives and preferences to play video games. In other words, we investigate players’ learning outcomes from playing non-educational games, and how these relate to wellbeing outcomes of playing, and gaming motivation.

Previous research has identified the potential of non-educational games in increasing learning motivation, enhancing cognitive performance, and learning social skills (e.g., Gee, 2003; Reinders, 2012; Silva et al., 2021). However, there have been relatively few empirical studies attempting to investigate what kinds of informal learning take place while playing non-educational games. Moreover, despite the extensive previous research on both wellbeing impact of games and gameplay motivation, which has been associated with wellbeing effects especially in the framework of the Self-Determination Theory (SDT), there is a lack of research that would investigate the motivational and wellbeing aspects of gaming together in relation to the informal learning in games.

Non-educational game is a term used in the context of game-based learning to describe games designed for entertainment as opposed to educational games designed for learning. Our focus is on informal, spontaneous learning occurring when voluntarily playing non-educational games. Informal learning, where learning takes place even when it is not the main objective of an activity, is an integral part of modern video game design, as games are designed to teach their players how to play them (*cf.*, Gee, 2003). However, we are interested in the diversity of informal learning taking place when playing non-educational games. Instead of asking how games can be used in teaching, we study the perceived learning outcomes of playing games.

In this study, this is essentially done in an open-ended manner, giving people the opportunity to describe what they have learned without presenting them with any presuppositions. The main goals of this study are (1) to form an empirically based understanding of what players perceive they have learnt from non-educational games, and (2) to statistically test how these learning outcomes relate to measurable wellbeing outcomes of playing, and formal assessments of gaming motivation.

Before formulating our research questions and hypotheses, we briefly address existing research in learning from non-educational games. Then we discuss the potential interconnections between the motivational constructs of learning and playing activities, and their relevance to human wellbeing.

## 2. Informal learning from games

Non-educational games, despite their name, have been frequently used for educational purposes (Squire, 2003; Wastiau et al., 2009; Martinez et al., 2022). Research on the use of commercial off-the-shelf games in education has identified their potential in learning diverse skills including dealing with depression (Olson, 2016), teamwork and other social skills (Sherry, 2016; Silva et al., 2021), attention abilities (Franceschini et al., 2013), and enhancing cognitive performance (Dale and Green, 2016). Furthermore, non-educational games have been used in teaching subjects such as languages (Chen and Yang, 2012; Reinders, 2012), history (Squire, 2005), and science (de Aldama and Pozo, 2020). Majority of previous research has studied learning outcomes using skill-testing or other forms of formal learning assessment. There have been surprisingly few empirical studies tackling the question of what kinds of informal learning take place while playing and how players themselves perceive their learning (Gee, 2003; Iacovides et al., 2014; Matijević and Topolovčan, 2019; *cf.* Scolari and Contreras-Espinosa, 2019). This research aims to take a more comprehensive approach by mapping out the variety of informal learning taking place in non-educational games.

### 2.1. Learning and the pursuit of wellbeing as possible underlying motives for playing games

From the developmental and evolutionary perspectives, human play and games have functioned as crucial forms of learning (e.g., Bruner et al., 1976). For children, play might be just a fun activity, but unknowingly children develop physical or social skills that are relevant in their future. In the literature, *playfulness* is characterized with qualities such as curiosity, openness to explore and engage with different contexts, and creative use of imagination (see a review in Masek and Stenros, 2021). Such characteristics of exploratory and experimental orientation to environment appear beneficial for individual development, if the term “development” is taken as an organism’s *actualizing tendency* toward maintaining and enhancing itself (Rogers, 1963 [2008]; see also Deci and Ryan, 1985). In general, one could argue playfulness having a role in the motivational attitude that fosters learning and self-enhancement. In particular, it is intriguing to conceive human play and playfulness within the framework of self-determined organismic growth. From this perspective, both the playful and “learnful” activities are seen to fulfill the basic needs of gaining experiences of autonomy, self-efficacy and social relatedness (Ryan and Deci, 2000). Therefore, according to the SDT, such actualizing tendencies not only should result in development, but through a genuine self-realization they should also constitute personal wellbeing (Ryan et al., 2008).

In the present study, we posit human development and wellbeing as being more or less intertwining constructs, both relating to organismic strive for “a good life” and being “fully functioning” in terms of optimal experience (Rogers, 1963 [2008]; Ryan and Deci, 2001). In the literature, there exist two principal views for defining wellbeing and conceptualizing what constitutes optimal functioning and experiences (see review in Ryan and Deci, 2001). Firstly, the hedonic view focuses on pleasure attainment (and pain avoidance), and the related desires and preferences of experiencing. The hedonic view on optimal experience, however, is not reducible to mere physical pleasures but more broadly relates to personal constructs about pleasure versus displeasure (Ryan and Deci, 2001, p. 143–145). Secondly, the eudaimonic view has its focus in doing things that are worth doing. From an eudaimonic perspective, activities yield wellbeing when they are in line with a person’s true self-realization, acknowledging that desires and preferred outcomes of (hedonically motivated) activity do not necessarily constitute wellbeing that is self-congruent (Ryan and Deci, 2001, p. 145–146). Self-determined human activity, satisfying the basic psychological needs of autonomy, competence, and relatedness, is usually associated with the concept of eudaimonia, and considered essential for intrinsic motivation, self-congruence, psychological growth, and psychological wellbeing (Ryan and Deci, 2001, p. 146–147).

Due to the close relationship of play and learning, it is tempting to consider the possible connections between the motives to play games and the learning outcomes yielded by gameplay. Student motivation is known to have a connection to learning outcomes (e.g., Janosz, 2012), but there is a lack of research on the relationship between gaming motivations and informal learning in games. Motivational models regarding gameplay usually only touch on learning and development. In such models, self-enhancement or learning as motives to play games often relate to the desire of becoming better in answering the game’s challenges or succeeding in competition (e.g., Sherry et al., 2006). In other words, they are limited to motives to become better at playing the game. However, few models of player motivation address a broader scope of learning. Demetrovics et al. (2011) Motives for Online Gaming Questionnaire (MOGQ) includes the Skill Development factor, concerning different sensorimotor and cognitive skills. Motives of the Autonomous Player (MAP) by Vahlo and Tuuri (submitted) is a model that incorporates a similar factor named Utility, referring to a motive to play games in order to train one’s brain and one’s memory and to develop oneself in general.

However, the somewhat marginal role of learning in the gameplay motivation models seems to be in conflict with the following argument by Crawford (1984), a game designer of the early era of video games in the seminal book “The Art of Computer Game Design”:

I claim that the fundamental motivation for all game-playing is to learn. This is the original motivation for game-playing, and it surely retains much of its importance. Game playing is a safe way to learn. The desire to learn, however, need not be conscious. Indeed, it may well take the form of a vague predilection to play games. Other motivations have little to do with learning and may assume greater local importance than the ancestral motivation to learn (Crawford, 1984, p. 13).

While it seems easy to agree with Crawford that learning is a fundamental motive to play games, it certainly does not appear to be a prominent theme in the current motivation research. A reason for such a discrepancy might be that people do not necessarily perceive their desire to learn in the form of explicit goal imagery (Schultheiss and Brunstein, 1999) and thus motives related to learning may retain an implicit or unconscious nature, as suggested by Crawford. With “other motivations” that “may assume greater local importance,” Crawford (1984, p. 13) refers to fantasy and exploration as well as competition and other social motives. Such a set of motives are also prevalent in many of the current models of gameplay motivation (see review in Vahlo and Tuuri, submitted).

It remains an empirically open question, to what degree learning and development implicitly relate to our desire to play games. In any case, we may indeed posit wellbeing (consisting of optimal experiences in both hedonic and eudaimonic terms) as a construct that inherently overlaps and interacts with both the general needs to play games and the general needs to develop and enhance ourselves.

### 2.2. Research questions and hypotheses

This paper consists of two substudies that correspond with the above-mentioned main goals of the study. The first substudy applies qualitative methodology in investigating what people consider to have (informally) learned by playing non-educational games (RQ1). We are also interested in identifying groups of informal learners in our sample (RQ2).

In the second substudy, we investigate how motivation and preferences to play games connect with the outlined informal learning outcomes of gameplay (RQ3a) and the outlined groups of informal learners (RQ3b). Similarly, we make an investigation of how wellbeing outcomes of gameplay connect with the learning outcomes (RQ4a) and the groups of learners (RQ4b).

Furthermore, in the second substudy, we distinguish self-attributed (eudaimonic) and gratification-based (hedonic) motives to play games and investigate how they connect with wellbeing outcomes of gameplay (RQ5) as well as, how they associate with informal learning outcomes (RQ6). For these questions, we have formulated two hypotheses. Firstly (H1), we expect that eudaimonic motives predict wellbeing outcomes and learning outcomes more strongly than hedonic motives. Secondly (H2), we also expect to find interactions between wellbeing outcomes and eudaimonic and hedonic motives that embody complementary functions of eudaimonic and hedonic activities in constituting wellbeing (e.g., Huta and Ryan, 2010).

## 3. Materials and methods

The research data (*N* = 1,202) was collected by using a survey targeted at adult (ages 18–70) UK and USA residents, who reported playing digital games at least occasionally. Participants were recruited through Prolific Academic Ltd., which is a private company that holds an online panel of 130,000 participants worldwide. Prolific is specialized in providing participants for different academic research tasks including surveys. The data was collected by applying Prolific’s option of balancing the sample between genders in both the UK and the US. From both countries, a total of 601 responses were collected, and the median time a user spent in taking the survey was approximately 18 min. All participants provided their written informed consent to participate in this study.

The survey consisted of three sections. In the first section survey participants responded to a short series of demographic questions. In the second section, the respondents were asked to specify their (1) reasons to play digital games, (2) gameplay preferences, and (3) their experienced wellbeing effects gained from playing digital games. Digital games referred to all games the participants had played on computers, consoles, and mobile devices. Stata 17.1/SE software was used for all quantitative analysis. In the final section of the survey, the participants were asked about their gaming habits and favorite games. They were also asked to describe in their own words what they had learned by playing games, and 95.2% (1,145) of the respondents answered this question. As the open-ended question about learning was presented at the end of the survey, the participants’ reminiscing focus on their learning did not generate any bias for answering the structured closed questions. After excluding irrelevant responses, which did not answer the question, there were 1,040 open-ended responses which were analyzed using data-driven content analysis to identify perceived learning outcomes.

A primary goal of the study was to investigate what players perceived they had learnt from non-educational games. The content analysis began with reading through each open response and placing it into one or more categories of learning outcomes. A data-driven approach, where categories were created as the analysis advanced, was used to ensure that all learning outcomes in the data were identified. This approach meant rereading the responses multiple times to include new categories in the analysis. In the next stage, a joint review of the learning outcomes was conducted by all authors and the learning outcome categories were grouped into main categories based on their similarities. An individual response could include learning outcomes from several main categories.

For studying gameplay motives, we used Motives for Autonomous Player (MAP) inventory, which is argued to be a general measure of gameplay motives that is applicable to all kinds of digital games, ranging from mobile puzzle games to massively-multiplayer online role-playing games (Vahlo and Tuuri, submitted). The MAP model incorporates nine factors, each representing a generic reason for playing games. Survey participants respond to the MAP inventory by selecting to what extent they agree or disagree that each motive item of the inventory accurately describes their reasons to play digital games (1 = Disagree completely, 7 = Agree completely). Since the validation study for the MAP model is still under review, we did an exploratory factor analysis for the combined data from the UK and the US to investigate the dimensionality of the 34-item inventory. The MAP inventory passed both the Kaiser-Meyer-Olkin (KMO) test (0.93) and The Bartlett test of sphericity (*Chi-square* = 22801.72, *df* = 561, *p* = 0.000) indicating that the inventory was suitable for a factor analysis. A parallel analysis (PA) test (Henson and Roberts, 2006) suggested a nine-factor solution, and therefore we extracted nine factors with promax rotation which does not assume factors to be orthogonal to each other. The factor model resulted in nine factors that can be described similarly to Vahlo and Tuuri (submitted) as *Immersive Agency* (4 items, *α* = 0.83, 95% CI from 0.81 to 0.84), *Competitive Mastery* (4 items, *α* = 0.84, 95% CI from 0.82 to 0.85), *Social* (4 items, *α* = 0.90, 95% CI from 0.89 to 0.91), *Addiction* (4 items, *α* = 0.87, 95% CI from 0.86 to 0.89), *Escapism* (4 items, *α* = 0.84, 95% CI from 0.82 to 0.85), *Utility* (3 items, *α* = 0.86, 95% CI from 0.85 to 0.87), *Affective Engagement* (4 items, *α* = 0.86, 95% CI from 0.84 to 0.87), *Boredom* (3 items, *α* = 0.76, 95% CI from 0.74 to 0.78), and *Nostalgia* (4 items, *α* = 0.88, 95% CI from 0.87 to 0.89). For the purposes of this study, we calculated both factor score variables and factor sum variables for all nine MAP factors by including all 34 items.

Among the MAP factors (see Supplementary Appendix 1), for the RQ5 and RQ6 of the study, *Immersive Agency, Competitive Mastery*, *Social*, and *Utility* were identified as *eudaimonic* motives, as all of them are self-attributive and relate to the three SDT needs (autonomy, competence and relatedness). Additionally, *Affective Engagement* was identified as *hedonic* motive, due to its straightforward focus on situated pleasure and gratification.

For producing a broad empirically-based understanding of informal learning in games and its connection to gameplay motives, we applied the gameplay activity inventory (GAIN) for measuring gameplay appreciation preferences (Vahlo et al., 2018). GAIN is a psychometrically validated 15-item inventory for assessing five dimensions in players’ preferences for videogame gameplay activities (1 = Very unpleasant, 7 = Very pleasant): *Aggression* (e.g., shooting, killing) *α = 0.83* (95% CI from 0.82 to 0.85), *Exploration* (e.g., gameplay exploration, character development) *α* = 0.75 (95% CI from 0.72 to 0.77), *Coordinate* (e.g., balancing movements, running and evading) *α* = 0.65 (95% CI from 0.61 to 0.68), *Caretaking* (e.g., choosing looks, dating) *α* = 0.73 (95% CI from 0.70 to 0.76), and *Management* (e.g., resource management) *α* = 0.65 (95% CI from 0.62 to 0.68). Factor sum variables for these five dimensions were calculated for the purpose of using them in statistical analyses of this study.

For studying wellbeing outcomes of gameplay, we applied a measure originally developed for assessing wellbeing effects of musical activity (WELLBEING), which is a 7-point Likert scale instrument (1 = Completely disagree, 7 = Completely agree). The original 36-item measure (Krause et al., 2018) identified five discrete dimensions: *mood and coping, esteem and worth, socialization, cognition*, and *self-actualization*. Since the items of this measure concern wellbeing outcomes in a manner that is not specified to musical context, applying the measure to a new activity was straightforward and only required minimal modifications to the items with respect to the updated framing of the main question, which was expressed as follows: “*Think about what kind of experiences you get from playing videogames. For each statement, choose the option that describes you the best. “Playing videogames…*.” We also shortened the measure to a 23-item version by using only the items demonstrating the strongest loadings to its respective factor in Krause et al. (2018) study. Since we made minor modifications to the inventory and as it has not been validated yet in research, we made an exploratory factor analysis (EFA) also on this measure (see Supplementary Appendix 2).

The 23-item version of the WELLBEING inventory clearly passed the KMO test (0.97) and The Bartlett test of sphericity (*Chi-square* = 23398.45, *df* = 253, *p* = 0.000), and the PA test suggested a three-factor solution (promax rotation). The first factor included items that Krause et al. (2018) argued to measure mood and coping as well as esteem and worth. We call this dimension *Mood and Coping* as these items had the highest loadings on the factor. The second factor included all items of the socialization dimension as well as an item from the self-actualization dimension. We call this factor *Social Connectedness*. Finally, items that loaded on the third factor consisted both of those of self-actualization and cognition. Since the former had higher loading than the latter, we name this factor *Self-Actualization*. It was not our intention to validate this measure in the current study, and therefore we did not omit any items that had low loadings. Instead, we generated factor score variables by using all of the 23 items and their loadings on all three factors. We also constructed factor sum variables by using only those items that showed a loading over 0.50 on the corresponding factor. The Cronbach alphas for these items on their factors were: *Mood and Coping* (6 items, *α* = 0.90, 9% CI 0. from 89 to 0.91), *Social Connectedness* (6 items, *α* = 0.96, 95% CI from 0.95 to 0.96), and *Self-Actualization* (5 items, *α* = 0.89, 95% CI from 0.88 to 0.90).

## 4. Results

### 4.1. Perceived learning outcomes

Our first task was to identify what the respondents perceived to have learnt by playing games (RQ1). Analyzing the experienced learning results yielded 117 subcategories of learning outcomes. Based on similarities between the subcategories, they were further categorized into 11 main categories (Table 1).

**Table 1:** Main categories of learning outcomes.

Main category	Freq.	Percent
Learning to play	103	9.2%
Learning about games	36	3.1%
Learning about self	91	8.4%
Thinking skills	451	54.2%
Interpersonal skills	236	24.5%
Embodied behaviors	236	25.0%
Subject matter	194	23.0%
Practical skills	236	23.4%
Coping skills	157	15.4%
Self-enhancement	156	14.1%
Learning to learn	18	1.5%

#### 4.1.1. Learning to play

The self-reported learning outcomes in this main category included mentions of learning to play the game the respondents were engaged with at any given time. Some responses disregarded this kind of learning, indicating they felt this was not a relevant skill, or not the kind of response the survey question was meant to bring.

I do not think I have learned anything from video games, apart from how to play the game in question. I think they are too limited and abstracted to be of any use in real life.

Learning to play also included mentions of gaining further understanding of game design or gaining skills applicable to any game in general or in a particular genre.

#### 4.1.2. Learning about games

Learning results described by the respondents also included knowledge of game cultures. This included game literacy, as in a deeper and broader knowledge of games and their fictional world, as well as knowledge of different game communities and their dynamics. There were also mentions of gaining knowledge of online communication and online cultures more broadly.

I’ve learned that people like to portray things within the games that they cannot in real life. They can be almost anything they want to be or do anything they want to do in a virtual world.

#### 4.1.3. Learning about self

Self-discovery through gaming was described by many respondents. They described learning about their own skills and attributes, like one respondent, who described:

I’ve learned how adaptable I am. How I react to certain situations as well as how I deal with failure.

In this category, there were also respondents mentioning how they learned about their own game preferences.

#### 4.1.4. Thinking skills

Thinking skills were the most common learning outcomes mentioned by respondents, with an emphasis on problem-solving, strategizing, management skills, decision making, creativity, and long-term planning.

Video games have taught me to be creative in using the character’s skills to solve challenging puzzles in areas of the game that involve the main story plot or side quests. For open world survival video games, it has taught me to manage time and resources to continue surviving and learning the game world’s harsh realities without real world consequences.

#### 4.1.5. Interpersonal skills

Different forms of collaboration and teamwork as well as communication skills were frequently mentioned by respondents as results of their gameplay experience. Some mentioned learning to relate to others particularly in a high stress competitive setting. Other commonly mentioned learning outcomes in this main category included an increased understanding of people in general and learning how to form relationships.

I have learned how to build team friendships and motivating fellow players and I have found I enjoy this.

#### 4.1.6. Embodied behaviors

The most common learning outcomes in this main category were hand-eye coordination, muscle memory, reaction time, and dexterity, and there are also responses mentioning motor skills generally. This main category also included mentions of increased spatial awareness and better pattern recognition. Some respondents also described using games for a therapeutic purpose, such as improving their dexterity after a surgery. Responses in this category were usually quite brief, such as:

My reactions are increased playing certain games.

#### 4.1.7. Subject matter

Respondents recounted gaining more knowledge of a diverse range of topics. Mentions of learning about history, mathematics and science, and different cultures were frequent, but there was significant diversity in the responses, including trivia knowledge on many topics such as football, weapons, or cars, to mention a few.

I have learned more about the rules of sports when playing games like Fifa.

#### 4.1.8. Practical skills

Language skills were the most common practical skill mentioned as a learning outcome. There were also frequent mentions of learning skills related to technology, economy, and creative writing. Some respondents described how they had used these skills in their everyday lives outside of gaming, for instance, their skills in interpreting maps and using them for navigation.

I now have a much firmer grasp of how maps translate into real world environments.

#### 4.1.9. Coping skills

Skills related to self-regulation were exhibited in the responses most commonly through perseverance, “how to keep trying until you are the best,” or as one respondent describes:

I have learnt patience and perseverance to keep going even if I feel like there is no hope. You never know when that good day is coming.

This category also included mentions of skills such as emotional or behavioral control, mood management, and stress management. Gaming as a form of positive escapism was another prominent learning outcome in this category.

#### 4.1.10. Self-enhancement

Some learning outcomes in the data, such as patience, determination, and flexibility, were related to attitudes or personality traits that are currently considered positive and worthy of pursuing and developing (in Western cultures). One respondent described learning, “[h]ow to be more patient, how to not rush at things and take your time to make the right moves.”

Gaining confidence through playing was also frequently mentioned, and some respondents described games and gaming as particularly meaningful, stating for example that “gaming has given me a sense of purpose.”

#### 4.1.11. Learning to learn

A small number of respondents also described they had gained learning skills through playing games. These mentions pertained to specific learning strategies or techniques such as research skills or note-taking or learning skills in general as in the following quotation.

Learned to pick out a certain skill or skillset and develop said skill(s) until they are mastered, or at least improved drastically.

### 4.2. Cluster analysis on informal game-based learning

The second task (RQ2) of this study concerned identifying groups of informal learners in our sample by applying cluster analysis. Based on the learning outcome categories derived from the data-driven analysis, we constructed 11 new dummy variables for each survey respondent in the data and assigned them a value of 0 or 1 based on whether their response included learning outcomes from the corresponding category. We report in Table 2 how many learning categories were mentioned by the survey participants of the survey.

**Table 2:** Distribution of survey respondents (*N* = 1,202) according to how many types of learning their answers represented.

Number of learning categories per a respondent	Freq.	Percent
0	162	13.5%
1	452	37.6%
2	378	31.4%
3	154	12.8%
4	41	3.4%
5	11	0.9%
6	3	0.2%
7	1	0.1%

**Figure 1:**
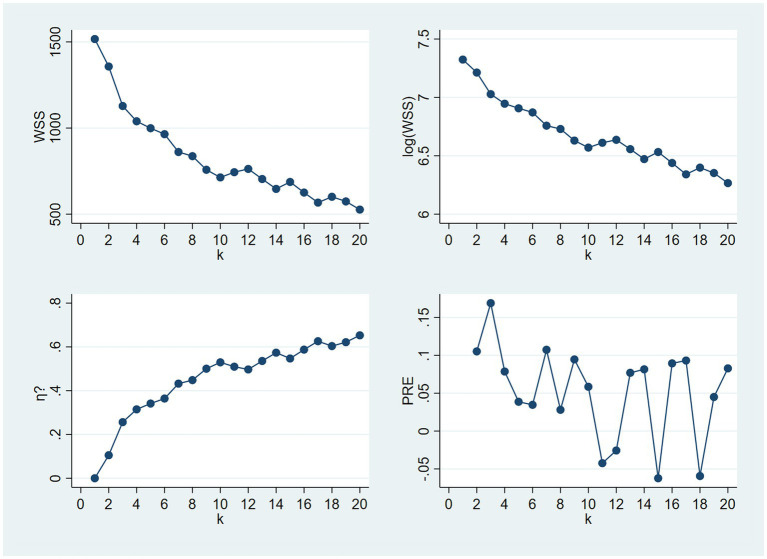
Scree plots for identifying the smallest justified number of clusters, based on which a 3-cluster solution was selected.

To study the relationships between different learning outcome categories on the level of respondents, we conducted an exploratory cluster analysis on the generated 11 dummy variables. To identify the appropriate number of clusters, we examined the scree plots created from the within-cluster sum of squares (WSS) and its logarithm [log(WSS)] for all solutions between 2 and 20 clusters (Makles, 2012; Figure 1). Both suggested a three-cluster solution, which we proceeded to generate.

An exploratory cluster analysis with Stata 17.1/SE software was then made with a three-cluster solution. The cluster analysis was conducted as an unsupervised K-Means clustering using the Jaccard index. The partitional clustering algorithm of K-Mean assigns *n* observations into non-overlapping clusters based on a predefined number of groups. K-Means is an iterative procedure that minimizes the total within-cluster variance while maximizing the variance between the clusters (Mehmetoglu and Venturini, 2021). The Jaccard coefficient is a procedure for calculating the similarity between two binary vectors based on the proportion of matches when the value of the included variables equals 1 (Hennig, 2007; Tan et al., 2021). The three clusters (Table 3) were constructed based on co-occurrences of the main learning categories in the data. The whole survey sample of 1,202 respondents was included in the cluster analysis.

**Table 3:** Results of cluster analysis, reporting the three groups of informal learning from games, cluster sizes, and proportions in the learning outcome main categories.

	Cluster A	Cluster B	Cluster C
	Learning perseverance	Learning practices and communalities	Learning to perform
*N*	323	278	601
Learning to play	2.2%	5.4%	13.5%
Learning about games	1.2%	3.2%	3.8%
Learning about self	1.9%	6.1%	11.3%
Thinking skills	0.0%	5.0%	72.7%
Interpersonal skills	0.0%	55.4%	13.6%
Embodied behaviors	0.3%	15.8%	31.8%
Subject matter	15.5%	21.2%	14.1%
Practical skills	0.0%	59.7%	11.6%
Coping skills	24.1%	8.3%	9.3%
Self-enhancement	17.6%	11.2%	11.3%
Learning to learn	1.9%	1.1%	1.5%
Age	32.3	29.6	31.0
Male	47.1%	51.8%	49.1%
Female	52.3%	43.6%	48.3%
Non-binary	0.3%	4.3%	2.6%
Not disclosed	0.3%	0.3%	0.0%

The three resulting clusters represent distinct types of learners, based on their learning outcomes (Table 3). Only the *Subject matter* learning category was relatively equally distributed across the clusters. The first cluster denotes learning outcomes relating to learners themselves and especially to the development of patience and perseverance in overcoming the challenges of life (*coping skills*, *self-enhancement*). The categories that differentiate the second cluster from the other two emphasize practical everyday skills that are often embedded in social communities (*practical skills*, *interpersonal skills*). The third cluster appears to represent performance-oriented cognitive and sensorimotor competence (*thinking skills*, *embodied behaviors*) that seems most closely related to the gameplay-situated, strategical, logical, and embodied skills needed for performing well in a game.

### 4.3. Connections of player motivations and preferences with learning

Next, we investigated how the identified 11 categories of informal learning from digital games were associated with gameplay motives and preferences (RQ3). This was studied by exploring how gaming motives and gameplay activity type preferences were related to learning (RQ3a) and whether learner types, i.e., the three clusters, differed from each other in what motivated players of these learner types to play games, and which gameplay activities they preferred (RQ3b). These analyses were done by applying the nine-factor Motives of the Autonomous Player (MAP) inventory, and the five-factor gameplay activity inventory (GAIN), and by relating both of these measures to (a) learning types and (b) learner types.

To perform these analyses, we applied factor sum variables for the 15-GAIN and the 34-MAP factors. For studying how the MAP motives to play digital games and the GAIN factors of gameplay preferences were associated with learning outcomes (RQ3a), we calculated multiple logistic regressions in which each of the learning outcome categories were assigned as the dependent outcome variable at a time. Since earlier research has indicated that motives to play digital games and gameplay activity type appreciation are related to each other, we decided to calculate the logistic regressions separately for the GAIN factors and the MAP factors to avoid possible multicollinearity issues. The results of the logistic regressions are reported in Table 4.

**Table 4:** Multiple logistic regression between gameplay preference factor sums, motive factor sums, age, and female gender and the dependent learning outcome variables.

Logistic regressions	Learn 1	Learn 2	Learn 3	Learn 4	Learn 5	Learn 6	Learn 7	Learn 8	Learn 9	Learn 10	Learn 11
*N*	103	36	91	451	236	236	194	236	157	156	18
Model 1: preferences											
Aggression	0.04	0.50**	−0.04	−0.09	0.13*	0.09	0.11	0.01	0.03	−0.08	0.08
Caretaking	0.10	0.11	−0.07	−0.04	0.00	−0.09	0.13	0.07	0.00	0.08	0.10
Coordinate	−0.01	−0.07	−0.06	−0.04	−0.06	0.11	−0.05	−0.10	0.11	0.08	0.42
Management	−0.02	−0.16	0.00	0.08	0.02	−0.05	0.00	0.05	−0.10	−0.04	0.02
Exploration	0.02	−0.02	0.18	0.20**	0.05	0.12	0.42***	0.19*	−0.03	0.16	−0.38
Age	0.01	0.02	0.01	−0.02**	−0.03***	0.01	−0.01	−0.02*	0.02**	0.02**	−0.03
Female	0.12	0.20	0.03	−0.18	−0.22	0.10	−0.48*	−0.40*	0.35	0.38	−0.41
Model 2: motives											
Imm.Agency	−0.10	0.05	0.02	0.07	0.05	−0.04	0.27**	0.14	−0.13	−0.25**	0.05
Nostalgia	0.21*	0.02	0.06	−0.02	−0.19**	0.03	0.03	−0.03	0.10	0.20*	−0.01
Social	−0.11	0.10	0.02	−0.02	0.56***	0.00	−0.11	0.00	−0.16*	−0.08*	0.03
Comp.Mast.	0.24*	0.25	0.02	−0.06	0.04	0.03	−0.05	−0.16*	0.08	0.02	0.30
Aff.Eng.	0.10	−0.05	−0.19	0.12	0.04	0.12	0.25*	0.14	−0.02	0.10	−0.75*
Utility	−0.13	−0.31*	−0.20*	0.21***	−0.05	0.19**	−0.03	0.19**	0.04	0.01	0.24
Escapism	−0.10	0.51*	0.22	−0.08	0.09	−0.11	−0.04	−0.08	0.24*	0.15	0.65*
Addiction	−0.07	−0.01	−0.01	−0.12*	−0.08	−0.06	−0.08	−0.01	−0.04	0.07	−0.21
Boredom	−0.09	−0.33*	−0.22*	0.06	0.00	−0.02	0.02	−0.06	0.00	0.10	0.00
Age	0.01	0.02	0.01	−0.02**	−0.01	0.00	−0.01	−0.02**	0.02*	0.03**	−0.03
Female	0.21	−0.02	0.04	−0.15	−0.11	−0.10	−0.55**	−0.35*	0.19	0.44	−0.44

Learn 1, Learning to play; Learn 2, Learning about games; Learn 3, Learning about self; Learn 4, Thinking skills; Learn 5, Interpersonal skills; Learn 6, Embodied behaviors; Learn 7, Subject matter; Learn 8, Practical skills; Learn 9, Coping skills; Learn 10, Self-enhancement; Learn 11, Learning to Learn. *p < 0.05, **p < 0.01, ***p < 0.001.

By observing the results of the logistic regressions, we can note that motives to play were more clearly associated with learning outcomes than gameplay preference factors. All of the nine motives to play digital games were associated with at least one type of learning. In comparison, only *Aggression* and *Exploration* of the five GAIN factors were associated with any type of induced learning.

Playing because of *Nostalgia* and *Competitive Mastery* were associated with the learning outcome *Learning to Play*. *Competitive Mastery* motive was found to be associated with only one additional learning outcome, that of *Practical Skills*, and this association was negative. Female gender was also found to be a negative predictor of the *Practical Skills* learning outcome whereas younger age and the *Utility* motive positively predicted this type of learning.

The *Utility* motive was positively associated also with *Embodied Behaviors*. No other independent variables were found to be associated with this type of learning. However, the *Utility* motive, together with the *Exploration* gameplay preference, predicted even more clearly the *Thinking Skills* type of learning. This type of learning was negatively predicted by the *Addiction* motive and higher age. Together with the *Boredom* motive, the *Utility* motive negatively predicted *Learning about Self*.

The gameplay preference for *Aggression* was a clear predictor for *Learning about Games* alongside with the *Escapism* motive and negatively with the *Boredom* and the *Utility* motives. *Aggression* preference predicted also *Interpersonal Skills* type of learning, although the main precedent for this learning type was clearly the *Social* motive. Furthermore, lower age and lower *Nostalgia* motive also predicted this learning category. The *Social* motive was a negative precedent for *Coping Skills* while *Escapism* and a higher age predicted this type of learning positively. Together with the *Immersive Agency* motive, the *Social* motive was furthermore associated negatively also with *Self-Enhancement*. This type of learning was positively predicted by higher age and the *Nostalgia* motive, which implies an inclusion of autobiographical, self-reflective engagement in regard to gameplay.

Preference in the gameplay activity *Exploration* predicted the learning type of *Subject Matter* together with gameplay motives of *Immersive Agency* and *Affective Engagement*. The female gender was associated with this type of learning negatively. Finally, lower score in *Affective Engagement* motive and higher in *Escapism* were found to be precedents for the *Learning to Learn* outcome. No other predictors for this final type of learning were found due to the small number of observations of this type.

Gaming motives, gameplay preferences, and demographic variables showed clear and versatile associations to the learning outcomes (RQ3a). As a next step in analysis, we examined if these learning associations could also be found on the level of learner types, that is, the constructed three learner clusters (RQ3b). In these analyses, factor sum variables for both the GAIN and the MAP factors were utilized, and factor means as well as standard deviations were calculated for the nine-factor MAP and five-factor GAIN constructs. We then did a series of one-way analysis of variance (ANOVAs) between the three clusters for motive and gameplay activity factors to identify if there were statistically significant differences between the group means.

A series of pairwise *t*-tests for significance and effect sizes (Cohen’s *d*) were next calculated to further analyze the statistically significant differences between the cluster means, as reported in Table 5. A pairwise *t*-test comparison of average motive sums between the three clusters revealed that participants of Cluster B were more motivated to play than those of Cluster C (*p* = 0.0071, Cohen’s *d* = 0.20, 95% CI from 0.05 to 0.34) and Cluster A (*p* = 0.0000, Cohen’s *d* = 0.50, 95% CI from 0.33 to 0.66). With the exceptions of *Addiction* and *Boredom*, there were statistically significant differences in all motive factors between the clusters. For Cluster A and Cluster B the effect sizes between the means were most notable in *Social* (Cohen’s *d* = 0.60, 95% CI from 0.43 to 0.76), *Immersive Agency* (Cohen’s *d* = 0.48, 95% CI from 0.32 to 0.64), *Utility* (Cohen’s *d* = 0.37, 95% CI from 0.21 to 0.54), and *Nostalgia* (Cohen’s *d* = 0.36, 95% CI from 0.20 to 0.52) in which Cluster 2 had clearly higher mean values than Cluster A. Cluster B and Cluster C were relatively similar to each other regarding their motive means. Yet the Welch *t*-test (one-sided) found statistically significant differences between these two clusters in *Social* (*p* = 0.0000, Cohen’s *d* = 0.33, 95% CI from 0.18 to 0.47), *Immersive Agency* (*p* = 0.0047, Cohen’s *d* = 0.21, 95% CI from 0.06 to 0.35), *Affective Engagement* (*p* = 0.033, Cohen’s *d* = 0.15, 95% CI from 0.01 to 0.30), and *Escapism* (*p* = 0.027, Cohen’s *d* = 0.16, 95% CI from 0.02 to 0.30) motives. In all of these cases, the mean values of Cluster B were higher than those of Cluster C.

**Table 5:** Motive and gameplay activity type factor sum means and standard deviations for three learner clusters.

Factor sums	Cluster A (*N* = 323)			Cluster B (*N* = 278)			Cluster C (*N* = 601)			Model
	Mean	*SD*	ANO	Mean	*SD*	ANO	Mean	*SD*	ANO	
Immersive agency	3.77	1.27		4.37	1.24	Ac	4.11	1.27	A	MAP
Competitive mastery	3.54	1.31		4.01	1.38	A	3.89	1.30	a	MAP
Affective engagement	5.58	1.03		5.97	0.88	A	5.84	0.86	A	MAP
Nostalgia	3.89	1.50		4.42	1.46	A	4.31	1.47	A	MAP
Utility	3.62	1.51		4.18	1.50	A	4.11	1.49	A	MAP
Social	3.70	1.58		4.64	1.57	AC	4.12	1.62	A	MAP
Addiction	2.43	1.23		2.65	1.31		2.50	1.37		MAP
Escapism	5.11	1.24		5.46	1.27	ac	5.27	1.07		MAP
Boredom	4.81	1.34		4.80	1.39		4.83	1.27		MAP
Aggression	4.19	1.58		4.69	1.49	A	4.48	1.61	a	GAIN
Caretaking	4.29	1.26		4.49	1.36		4.38	1.31		GAIN
Coordinate	4.33	1.30		4.33	1.35		4.40	1.25		GAIN
Management	4.45	1.17		4.66	1.17	a	4.60	1.17		GAIN
Exploration	5.38	1.16		5.71	1.10	A	5.66	1.08	A	GAIN
Average, motives	4.05	0.92		4.50	0.90	Ac	4.33	0.85	A	
Average GAIN	4.53	0.88		4.78	0.91	A	4.70	0.81	a	

One-way analyses of variance (ANOVAs) between the clusters means: a/c = *p* < 0.05, A/C = *p* < 0.001 in which the alphabets indicate between which two clusters there is a statistically significant difference. Cluster A, Learning perseverance; Cluster B, Learning practices and communalities; Cluster C, Learning to perform.

As for the five types of gameplay activity preferences, only *Aggression* and *Exploration* sums differed between the clusters. Cluster A had lower *Aggression* preference than both Cluster B (Cohen’s *d* = 0.32, 95% CI from 0.16 to 0.49) and Cluster C (Cohen’s *d* = 0.18, 95% CI from 0.05 to 0.32). The same was true for the *Exploration* sum between Cluster A and Cluster B (Cohen’s *d* = 0.29, 95% CI from 0.13 to 0.45) and Cluster A and Cluster C (Cohen’s *d* = 0.25, 95% CI from 0.12 to 0.39).

### 4.4. Connections of wellbeing outcomes with learning

As with the analyses made on gameplay appreciation and gaming motives, we investigated how wellbeing outcomes of gameplay are connected with the learning outcomes (RQ4a) and the groups of learners (RQ4b). For this purpose, we applied the three wellbeing factor sum variables that were constructed after making an exploratory factor analysis on the 23-item version of the WELLBEING inventory. Analogously to the model reported above, we calculated multiple logistic regressions in which the 11 learning dummy variables were set as dependent outcome variables one by one, and the three factors of *Identity Actualization*, *Social Connectedness*, and *Mood and Coping* were assigned as independent predictors, accompanied again by age and female gender. The results of these regressions are reported in Table 6.

**Table 6:** Multiple logistic regression between wellbeing factor sum variables, age, and female gender and the dependent learning outcome variables.

Logistic regressions	Learn 1	Learn 2	Learn 3	Learn 4	Learn 5	Learn 6	Learn 7	Learn 8	Learn 9	Learn 10	Learn 11
*N*	103	36	91	451	236	236	194	236	157	156	18
Identity actualization	−0.12	−0.01	0.31**	0.15*	−0.15	0.04	0.01	0.12	0.11	0.07	0.22
Social connection	0.08	0.24	−0.14	−0.09	0.62***	−0.08	−0.08	−0.06	−0.16*	−0.11	−0.06
Mood and coping	0.08	−0.04	−0.24*	0.11	0.05	0.08	0.40***	0.10	0.21*	0.21*	−0.15
Age	0.01	0.02	0.01	−0.01*	−0.01	0.00	−0.01	−0.02*	0.02**	0.02**	−0.03
Female gender	0.14	−0.13	0.03	−0.10	−0.11	−0.16	−0.59***	−0.34*	0.27	0.48**	−0.45

Learn 1, Learning to play; Learn 2, Learning about games; Learn 3, Learning about self; Learn 4, Thinking skills; Learn 5, Interpersonal skills; Learn 6, Embodied behaviors; Learn 7, Subject matter; Learn 8, Practical skills; Learn 9, Coping skills; Learn 10, Self-enhancement; Learn 11, Learning to Learn. *p < 0.05, **p < 0.01, ***p < 0.001.

All the three wellbeing factors were found to be associated with learning outcomes, but only six of the learning types were related to wellbeing in a statistically significant fashion. From the wellbeing dimensions, *Identity Actualization* was associated with *Learning about Self* and *Thinking Skills*. The wellbeing factor *Social Connectedness* was positively related to *Interpersonal Skills* type of learning, and negatively to *Coping Skills*. The third wellbeing factor *Mood and Coping* was a positive precedent for *Subject Matter*, *Coping Skills*, and *Self-Enhancement* types of learning, and a negative precedent for *Learning about Self*. These logistic regressions were followed again by group comparisons between the three learner clusters and their factor sum means. Table 7 reports these comparisons.

**Table 7:** Wellbeing factor sum means and standard deviations for three learner clusters.

	Cluster A (*N* = 323)			Cluster B (*N* = 278)			Cluster C (*N* = 601)		
	Mean	*SD*	ANO	Mean	*SD*	ANO	Mean	*SD*	ANO
Identity actualization	3.77	1.27		4.37	1.24	Ac	4.11	1.27	A
Social connectedness	3.54	1.31		4.01	1.38	AC	3.89	1.30	A
Mood and coping	5.58	1.03		5.97	0.88	Ac	5.84	0.86	A
Average, wellbeing	4.05	0.92		4.50	0.90	AC	4.33	0.85	A

One-way analyses of variance (ANOVAs) between the clusters means: a/b/c = *p* < 0.05, A/B/C = *p* < 0.00 in which the alphabets indicate between which two clusters there is a statistically significant difference.

There were several statistically significant differences between the three learner clusters and their wellbeing factor sum means, as indicated by ANOVAs (Table 7). As in the group comparisons made between gaming motives and gameplay appreciation factors, Cluster B had the highest values also for the experienced wellbeing, across all three of its factors.

Next, we did a series of pairwise *t*-tests for significance and effect sizes (Cohen’s *d*) between the wellbeing factor sum means of the three clusters. In the case of all of the three wellbeing factors, participants of both Cluster B and Cluster C reported clearly higher wellbeing than participants of Cluster A. These differences were the most drastic between Clusters A and B in *Social Connectedness* (*p* < 0.000, Cohen’s *d* = 0.61, 95% CI from 0.45 to 0.78), *Identity Actualization* (*p* < 0.000, Cohen’s *d* = 0.53, 95% CI from 0.36 to 0.69), and *Mood and Coping* (*p* < 0.000, Cohen’s *d* = 0.42, 95% CI from 0.26 to 0.58). Also, Cluster B and a clearly higher mean than Cluster C in *Social Connectedness* (*p* < 0.000, Cohen’s *d* = 0.36, 95% CI from 0.21 to 0.50).

The analyses of this study have indicated that particular forms of gameplay appreciation and especially self-attributed motives to play are associated with game-based informal learning. Furthermore, we have seen that perceived wellbeing outcomes are also related to specific types and ways of learning. What remains unexplored, is how the motives that are associated with learning should be conceptualized, and how gaming motives and wellbeing as an outcome of gameplay are related to each other on a more general level. These latter themes are investigated in this last part of the present study, in which we distinguish self-attributed (eudaimonic) and gratification-based (hedonic) motives to play games and investigate how these two types of motives connect with wellbeing outcomes of gameplay (RQ5) as well as how they associate with informal learning outcomes (RQ6). Our hypotheses are that eudaimonic motives predict the wellbeing and learning outcomes more strongly that hedonic motives (H1), and that eudaimonic and hedonic motives to play games embody complementary functions in the constitution of wellbeing outcomes (H2).

### 4.5. Eudaimonic and hedonic gameplay motives

The analyses of this study have indicated that particular forms of gameplay appreciation and especially self-attributed motives to play are associated with game-based informal learning. Furthermore, we have seen that perceived wellbeing outcomes are also related to specific types and ways of learning. What remains unexplored, is how the motives that are associated with learning should be conceptualized, and how gaming motives and wellbeing as an outcome of gameplay are related to each other on a more general level. These latter themes are investigated in this last part of the present study, in which we distinguish self-attributed (eudaimonic) and gratification-based (hedonic) motives to play games and investigate how these two types of motives connect with wellbeing outcomes of gameplay (RQ5) as well as how they associate with informal learning outcomes (RQ6). Our hypotheses are that eudaimonic motives predict the wellbeing and learning outcomes more strongly that hedonic motives (H1), and that eudaimonic and hedonic motives to play games embody complementary functions in the constitution of wellbeing outcomes (H2).

**Figure 2:**
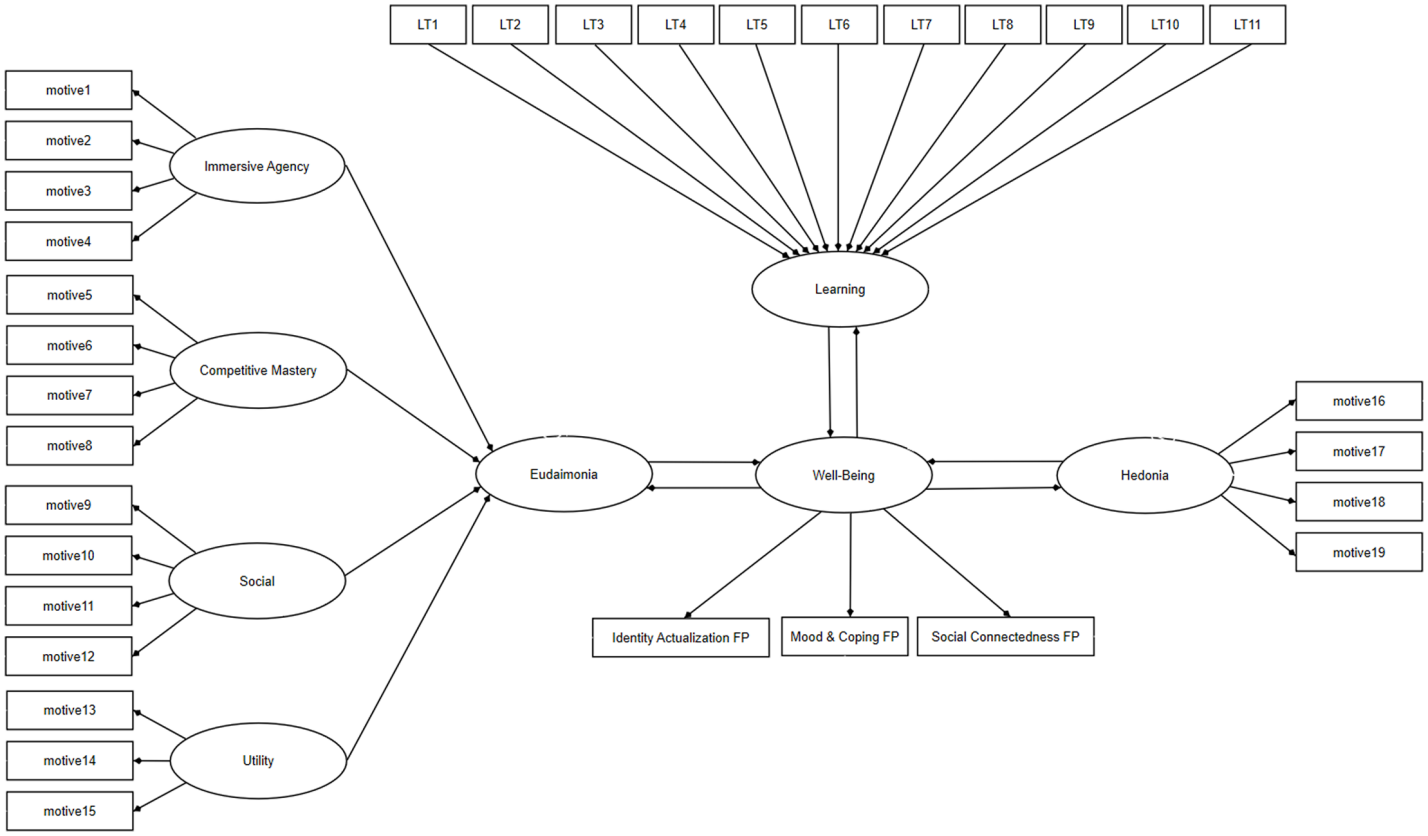
The PLS-SEM model by which the connections between eudaimonic and hedonic motives, wellbeing, and learning was explored. Manifest variables or indicators are reported as rectangles, and latent variables as ellipses.

The hypotheses (H1, H2) concerning the eudaimonic and hedonic gaming motives’ ability to predict wellbeing and learning outcomes of gameplay were tested by the means of partial-least squares structural equation modeling (PLS-SEM). In the model reported in Figure 2, the gameplay motive factors of *Immersive Agency, Competitive Mastery*, *Social*, and *Utility* were taken to be precedents of *Eudaimonic* gaming orientation whereas the gameplay motive factor of *Affective Engagement* was considered to represent a more *Hedonic* approach to gaming. Furthermore, all of the 11 learning categories were assigned to be precedents of *Learning*. All of the statistical analyses were made with Stata/SE 17.0, by making use of the *plssem* package by Mehmetoglu and Venturini (2019).

The main purpose of PLS-SEM is to explain as much of the variance of the dependent variable as possible. In contrast to covariance-based structural equation modeling (CB-SEM), PLS-SEM is considered to be an explorative technique for predicting and explaining associations between independent and dependent latent and observed variables in complex models (Hair et al., 2017; Mehmetoglu and Venturini, 2021). Furthermore, in PLS-SEM the measurement error of indicators is ignored whereas theory-driven confirmatory CB-SEM takes error variances of indicators into account.

In the PLS-SEM model (Figure 2), we have assigned *Wellbeing* as the dependent or endogenous composite (latent construct of weighted sums of the assigned indicators), measured by three factor point manifest variables of *Identity Actualization*, *Mood and Coping*, and *Social Connectedness*. For the three WELLBEING factors, we applied factor score variables instead of factor sums as the WELLBEING inventory has not yet been validated. Computing factor score variables after an EFA and including these variables in a PLS-SEM model is furthermore a recommended practice, as factor score variables represent how all included inventory items load on each extracted factor in the analyzed data (Mehmetoglu and Venturini, 2021). In the PLS-SEM model, *Immersive Agency*, *Competitive Mastery*, *Social*, *Utility, Affective Engagement*, and *Wellbeing* are all assigned as reflective models whereas *Learning* and *Eudaimonia* are constructed as formative models. In contrast to reflective models which assume that the indicators are treated as factors that measure a common underlying latent construct, in formative models each manifest variable is taken to be a predictor of the construct they are associated with. Reflective models were applied in the above-mentioned cases of gaming motives as these constructs are validated in prior research by confirmatory factor analyses and CB-SEMs (Vahlo and Tuuri, submitted).

Table 8 summarizes standardized factor loadings for the constructs included in the PLS-SEM model. The loadings of all reflective models are high, which is also reflected in the corresponding scale reliability scores. The formative model of *Eudaimonia* indicates that all included self-attributed motives were clear precedents of the construct. Items that load on *Immersive Agency* and *Social* had a stronger effect on *Eudaimonia* than those that loaded on *Competitive Mastery* and *Utility*.

**Table 8:** Standardized loadings of the PLS-SEM measurement model.

	Reflective:	Reflective:	Reflective:	Reflective:	Reflective:	Formative:	Reflective:	Formative
	Wellbeing	ImmAgenc	CompMast	Social	Utility	Eudaimonia	Hedonia	Learning
Identity actualization	0.93							
Social connect.	0.87							
Mood and coping	0.84							
Motive 1		0.82						
Motive 2		0.82						
Motive 3		0.77						
Motive 4		0.83						
Motive 5			0.82					
Motive 6			0.82					
Motive 7			0.83					
Motive 8			0.81					
Motive 9				0.88				
Motive 10				0.81				
Motive 11				0.89				
Motive 12				0.93				
Motive 13					0.88			
Motive 14					0.90			
Motive 15					0.87			
Motive 1						0.71		
Motive 2						0.74		
Motive 3						0.62		
Motive 4						0.71		
Motive 5						0.52		
Motive 6						0.52		
Motive 7						0.56		
Motive 8						0.56		
Motive 9						0.75		
Motive 10						0.61		
Motive 11						0.78		
Motive 12						0.78		
Motive 13						0.51		
Motive 14						0.57		
Motive 15						0.50		
Motive 16							0.81	
Motive 17							0.86	
Motive 18							0.84	
Motive 19							0.85	
Learning 1								−0.01
Learning 2								0.13
Learning 3								−0.07
Learning 4								0.32
Learning 5								0.83
Learning 6								0.02
Learning 7								0.33
Learning 8								0.28
Learning 9								0.01
Learning 10								0.04
Learning 11								0.05
Cronbach’s alpha	0.85	0.83	0.84	0.90	0.86		0.86	
DG	0.91	0.89	0.89	0.93	0.92		0.91	
rho_A	0.85	0.83	0.84	0.91	0.87	1.00	0.87	1.00

Average *R*^2^ = 0.51, Relative Goodness-of-Fit: 0.92. Weighting Scheme: Path. *N* = 1,202.

In the case of the *Learning* composite, the 11 learning categories had very different associations with the latent outcome of *Learning*. The learning category of *Interpersonal Skills* (Learning 5) had clearly the strongest effect on *Learning*. Also *Thinking Skills* (Learning 4), *Subject Matter* (Learning 7), and *Practical Skills* (Learning 8) had a notable association with *Learning*. It is important to emphasize that *Learning* of the PLS-SEM of this study is a summated composite, and that the 11 learning categories are assigned as potential predictors of it. This means that *Learning* is the diversity and versatility of informal learning, i.e., how multidimensional the learning from games is. In other words, the learning type of *Interpersonal Skills* is the strongest precedent for the versatility of game-based learning. In contrast to this, for instance, *Learning about Self* (Learning 3) and *Learning to Play* (Learning 1) were not associated with *Learning* which means that these types of learning do not predict that game-based learning would be multi-faceted.

Next, we continued to investigate discriminant validity of the PLS-SEM model by calculating the average variance extracted (AVE) for the reflective constructs *Wellbeing*, *Immersive Agency*, *Competitive Mastery*, *Social*, *Utility*, and *Hedonia* (*Affective Engagement*). To support discriminant validity for each composite, AVE for each reflective construct should be over 0.50 and higher than the square of the correlation between that construct and other reflective constructs included in the model (Fornell and Larcker, 1981; Farrell, 2009). The composites included in the study fulfilled these criteria. The average variance test results are reported in Table 9.

**Table 9:** Discriminant validity of the PLS-SEM model.

	Wellbeing	Immersive agency	Competitive mastery	Social	Utility	Formative: eudaimonia	Hedonia	Formative: learning
Wellbeing	1.00	0.47	0.28	0.44	0.22	0.64	0.27	0.09
Immersive agency	0.47	1.00	0.20	0.25	0.20	0.74	0.22	0.04
Competitive Mastery	0.28	0.20	1.00	0.26	0.28	0.44	0.09	0.02
Social	0.44	0.25	0.26	1.00	0.15	0.71	0.10	0.08
Utility	0.22	0.20	0.28	0.15	1.00	0.35	0.08	0.02
Eudaimonia	0.64	0.74	0.44	0.71	0.35	1.00	0.22	0.08
Hedonia	0.27	0.22	0.09	0.10	0.08	0.22	1.00	0.03
Learning	0.09	0.04	0.02	0.08	0.02	0.08	0.03	1.00
AVE	0.77	0.66	0.67	0.77	0.79		0.71	

Squared interfactor correlation vs. Average variance extracted (AVE).

Tables 10, 11 report the structural model of the PLS-SEM (Figure 2). All effects with the exception of the effect of *Wellbeing* on *Eudaimonia* were found to be statistically significant on the level *p* < 0.001, while the effect of *Wellbeing* on *Eudaimonia* was statistically significant on the level of *p* < 0.01. The model explained 67 percent of the variance of *Wellbeing*, almost all of the variance of *Eudaimonia*, approximately 27 percent of the variance of *Hedonia*, and only 9% of *Learning*. Regarding *Wellbeing*, the model explained between moderate and substantial amounts of its variance (Hair et al., 2021).

**Table 10:** Structural model of the PLS-SEM model.

Variable	Wellbeing	Eudaimonia	Hedonia	Learning
Wellbeing		0.018**	0.517***	0.301***
Immersive agency		0.511***		
Competitive mastery		0.125***		
Social		0.468***		
Utility		0.113***		
Eudaimonia	0.693***			
Hedonia	0.178***			
Learning	0.075***			
Adjusted *R*2	0.666	0.997	0.267	0.09

Standardized path coefficients (Bootstrap). *p < 0.05, **p < 0.01, ***p < 0.001.

**Table 11:** Direct, indirect, and total effects of the manifest variables and latent constructs on endogenous constructs of Wellbeing, Learning, Eudaimonia, and Hedonia.

Direct, indirect, and total effects	Direct	Indirect	Total
Wellbeing - > Eudaimonia	0.018	0.003	0.021
Wellbeing - > Hedonia	0.517	0.075	0.592
Wellbeing - > Learning	0.301	0.044	0.345
Eudaimonia - > Wellbeing	0.693	0.101	0.794
Eudaimonia - > Hedonia		0.409	0.409
Eudaimonia - > Learning		0.239	0.239
Hedonia - > Wellbeing	0.178	0.026	0.204
Hedonia - > Eudaimonia		0.004	0.004
Hedonia - > Learning		0.061	0.061
Learning - > Wellbeing	0.075	0.011	0.085
Learning - > Eudaimonia		0.002	0.002
Learning - > Hedonia		0.044	0.044
Immersive agency - > Wellbeing		0.405	0.405
Immersive agency - > Eudaimonia	0.511	0.007	0.518
Immersive agency - > Hedonia		0.209	0.209
Immersive agency - > Learning		0.122	0.122
Competitive mastery - > Wellbeing		0.099	0.099
Competitive mastery - > Eudaimonia	0.125	0.002	0.127
Competitive mastery - > Hedonia		0.051	0.051
Competitive mastery - > Learning		0.030	0.030
Social - > Wellbeing		0.370	0.370
Social - > Eudaimonia	0.468	0.007	0.474
Social - > Hedonia		0.191	0.191
Social - > Learning		0.112	0.112
Utility - > Wellbeing		0.089	0.089
Utility - > Eudaimonia	0.113	0.002	0.114
Utility - > Hedonia		0.046	0.046
Utility - > Learning		0.027	0.027

Standardized (*β*) path coefficients revealed that *Eudaimonia* had a substantial direct effect on *Wellbeing*, and from Table 11 we can furthermore note that *Eudaimonia* also had an indirect effect on *Wellbeing* making the total effect strong (*β* = 0.79). *Hedonia* also had a significant effect (*β* = 0.20) on *Wellbeing*, although this effect was rather weak in comparison to that of *Eudaimonia*. In regard to wellbeing, these results give clear support to the H1. From the motive factors associated with *Eudaimonia*, the motive factor *Immersive Agency* had the strongest indirect effect on *Wellbeing* (*β* = 0.41). In addition to the *Eudaimonia* motive, also the *Social* motive had a moderate indirect effect on *Wellbeing* (*β* = 0.37). In terms of learning outcomes of gameplay, *Eudaimonia* had a moderate indirect effect on *Learning* (*β* = 0.24), which however is much greater in comparison to the minor effect of *Hedonia* on *Learning* (*β* = 0.06). This further provides support to the H1 also in regard to informal learning outcomes. The effect of versatility of game-based informal *Learning* on *Wellbeing* was very weak (*β* = 0.08), albeit still statistically significant on the level of *p* < 0.001.

We furthermore included reversed investigations into whether *Wellbeing* outcomes from gaming were associated with the gaming motivations of *Eudaimonia* and *Hedonia*, and with informal game-based *Learning*. It was found that *Wellbeing* outcomes strongly predicted *Hedonia* (*β* = 0.59), thus the participants’ higher score in wellbeing assessment clearly was a precedent for hedonic motivation to play digital games. However, this was not the case with *Eudaimonic* motivation to play digital games, which interestingly was not predicted by *Wellbeing* outcomes (*β* = 0.02). Finally, it was also revealed that *Wellbeing* was moderately associated with *Learning* (*β* = 0.35).

In all, our results provide support for the hypothesized imbalanced effects of *Eudaimonic* and *Hedonic* motives on *Wellbeing* and *Learning*. The oppositely imbalanced results of the reversed investigations of *Wellbeing* as a predictor for *Eudaimonia* and *Hedonia* give support to our second hypothesis (H2). Hence, based on the results, one may indeed argue that eudaimonia and hedonia serve different, but possibly complementary functions in the constitution of a person’s wellbeing. This matter is further discussed in the following section.

## 5. Discussion

The outcomes of informal learning in this study are based on our analysis of players’ own perceptions of learning from games. We identified 11 main categories of learning outcomes. To identify potential learner types, we conducted a cluster analysis. Together with comparisons between these three learner types (Learning perseverance, Learning practices and communalities, and Learning to perform) it was revealed that players do differ from each other in what they articulate they have learned by playing games of their choice. Each of the three clusters denoted different profiles of learning, respectively emphasizing the specific areas of self-development, improving communal practices, and improving performative abilities in terms of both cognitive and behavioral skills. It is tempting to reflect on this result from the perspective of the SDT (Ryan and Deci, 2000), especially in terms of the three basic needs that constitute positive growth and motivation of individuals. Thus, it seems possible to make obvious linkages between the needs of Autonomy, Relatedness, and Competence, and the respective clusters of Learning perseverance (supporting autonomic self-development), Learning practices and communalities (supporting social relatedness), and Learning to perform (supporting the development of competence). This kind of interpretation would, however, require future studies focusing particularly on the prospect of this promising observation.

Secondly, the three learner types did have distinctive profiles, not only regarding the experienced learning outcomes but also player motives and preferred gameplay activity types. The main result of these comparisons was that the Learning perseverance (A) type of player-learners were notably less motivated to play games than the other two learner types, yet they enjoyed gameplay activities of coordination, caretaking, and management similarly to the Learning practices and communalities (B), and the Learning to perform (C) player-learners. The Learning practices and communalities player-learners differed from the Learning to perform player-learners in a much more subtle way, mostly regarding the Social motive and the Immersive Agency motive.

Our results indicated that playing because of social and immersive experiences (i.e., motives that were emphasized within the cluster B of player-learners), are associated with a high eudaimonic motivation to play games and, importantly, also with learning outcomes related to interpersonal and practical skills (e.g., language skills, teamwork, subject matter). Furthermore, we found that another, highly motivated player-learner type (i.e., the cluster C) that was not as motivated by social and immersive experiences reported learning outcomes that were closely associated with performance, competence, and honing skills by overcoming game challenges. The less motivated type of player-learners (i.e., the cluster A) perhaps considers games more as a method for self-development, self-enhancement, and coping, and thus has a more instrumental relationship with game experiences than the other two learner types.

On the level of gameplay activity types, it seems that game mechanics and dynamics that enable aggressive (e.g., shooting, killing) and explorative gameplay (e.g., character development, narrative progression) are attractive to players who (report to) have learned practical skills, interpersonal skills, thinking skills, and embodied behaviors by playing non-educational games (i.e., clusters B and C). For the player-learners of the cluster A, the aggressive and explorative gameplays are not similarly attractive, which means that this learner type has more balanced gameplay preferences. Perhaps this is another indicator of them being more focused on self-development, rather than the game and the social interactions it may enable. Related to this, it should be asked whether perceived learning and psychological need satisfaction, as argued in the SDT framework, are somehow related to each other. Our results seem to indicate that game-based learning might be closely associated with user gratification and satisfaction that players derive from games.

**Figure 3:**
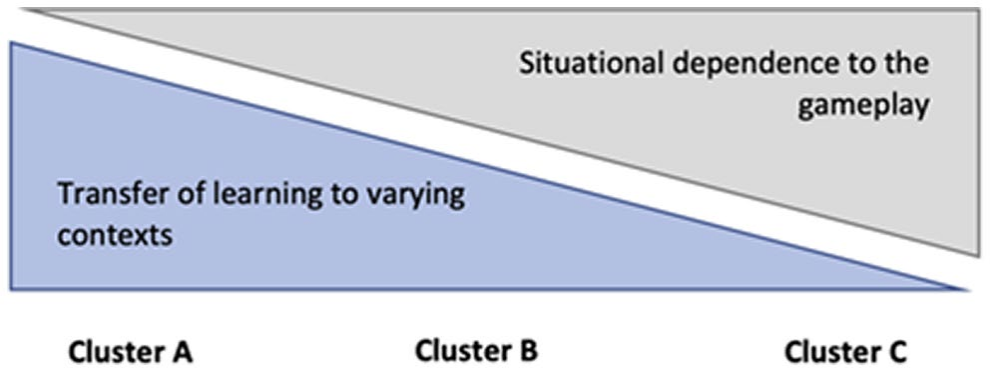
The two-way continuums of the transfer of learning and the dependence of learning in relation to the three clusters of learning outcomes.

Given that all three learner types outlined stem from voluntary play of non-educational games, it is interesting to note how each cluster varies in terms of how dependent the learning outcomes are on gameplay (Figure 3). Most notably, the Learning to perform cluster (C) seems to incorporate the closest situational dependence to the engagement with gameplay activities and “doing well” in answering the game’s challenges. This interpretation is underlined by the fact that this cluster included the largest amount of learning outcomes that were explicitly about learning to play. On the other end of the continuum, Learning perseverance cluster (A) seems to manifest skills that are most transferable to different contexts of everyday life, while also bearing the least number of skills with direct dependence to actual gameplay (e.g., strategizing, sensorimotor skills). The cluster B, Learning practices and communalities, is positioned between the other two, as it seems to both denote practical and interpersonal skills that are highly transferable while also associating with more gameplay-dependent similar skills as in the case of the third cluster.

In addition to analyzing the results from the perspective of the three player-learner clusters, we made a series of logistic regressions between gameplay preference factors, self-attributed motives, dimensions of wellbeing, and the outcome binary variables of the 11 types of informal learning. Multiple logistic regressions between gameplay preference factors, self-attributed gaming motives, and learning outcomes revealed that motives were more comprehensively and versatilely connected with learning than gameplay activity type preferences. A preference for *Exploration* clearly predicted Subject Matter type of learning outcome, and a preference for *Aggression* was related to Learning about Games. In comparison, all of the nine motive factors were associated with at least one learning outcome. For instance, the *Social* motive was a main precedent for Interpersonal Skills type of learning, the latter of which was the defining type of learning for the player-learner cluster B that was found to be the most highly motivated to play digital games.

The connections between gameplay activity type preferences and learning can perhaps be partly explained by considering what types of games emphasize *Exploration* and *Aggression* in their player-game interaction. *Exploration* covers player activities such as gameworld and story exploration, and character development and customization (Vahlo et al., 2018). These activities are frequent for role-playing games and action-adventure games, and both of these genres have many games that have rich story-driven and world-building qualities that can foster Subject Matter type of learning. Many games that have core gameplay loops based on *Aggression* are often also multiplayer online games of genres such as battle royale and MOBA (multiplayer online battle arena). In such genres, it is essential for players to understand game communities, and to have in-depth understanding about features of the game of their choice. Again, it is sensible that activities based on *Aggression* would be related to Learning about games.

Similarly to motives to play digital games, also all dimensions of wellbeing were found to be related with informal learning categories. Most noteworthy connections were found between the wellbeing factor of *Social Connectedness* and Interpersonal Skills type of learning, *Mood and Coping* factor and Subject Matter type of learning, and *Identity Actualization* and Learning about Self. The associations between *Social Connectedness* type of wellbeing and Interpersonal Skills type of learning as well as *Identity Actualization* and Learning about Self are rather self-explanatory, although it is noteworthy and interesting as a result that the connection between wellbeing and learning gained from playing is so clear. However, the relationship between *Mood and Coping* and Subject Matter type of learning is not as evident. How exactly do the optimal experiences of emotional self-regulation and coping with everyday issues function as a precedent for learning about Subject Matter? Further research is required for investigating this question.

A series of PLS-SEM analyses were done to study general questions about how eudaimonic and hedonic motives are related to the wellbeing outcomes of gaming and the versatility of game-based learning, and how learning and wellbeing furthermore are related to gaming motives. Importantly, these analyses showed that eudaimonic gaming motives (*Immersive Agency, Social, Competitive Mastery*, and *Utility*) were strong and important precedents for wellbeing effects of gaming, and also a significant precedent for game-based learning. Yet, the analyses also revealed that wellbeing from gaming was not a precedent for eudaimonic motivation to play digital games. Instead of that, wellbeing predicted the hedonic gaming motive (*Affective Engagement*) rather strongly.

These results raise two questions, both of which would be important to examine in further research. Firstly, we can consider that the etiology of eudaimonic motivation is probably not based on immediate gratification derived from an ongoing experience but rather, as the term indeed suggests, on the more profound values and virtues of the participating individual. According to the findings of Huta and Ryan (2010) there is a reason to believe that hedonia and eudaimonia co-constitute wellbeing at different time scales, the former relating to more immediate outcomes and the latter relating to longer and person-level outcomes of activities. Hence, immediate experiences are likely to have an effect on the hedonic motives to return or not to return to play a game, but the underlying eudaimonic motives would not be similarly affected by it. Secondly, the strong effect of eudaimonic self-attributed motives to play digital games makes us ask what factors outside the immediate gaming experience affect the eudaimonic motivation, and how game developers and other stakeholders could put forward services and solutions that are able to build eudaimonic motivation. Future research should focus on investigating to what extent this kind of effect could be achieved through game design practices that support social interaction and immersive agency, the two motives which had the most significant effect on both wellbeing and learning. Regarding the issue of supporting eudaimonic motives to play by design practices, it is furthermore important to consider in future research how prevalent game challenge types are associated with the identified 11 learning types and especially eudaimonic motives to play digital games (Vahlo and Karhulahti, 2020).

One of the prominent limitations of the present study relates to the nature of gathering all of the data with a single survey. Therefore, instead of adopting a longitudinal methodological approach, only a single point of measurement was used for investigating different phases of the process of motivational development and the outcomes of gameplay. Another limitation of this study is related to combining the 11 learning categories that were identified as a result of the qualitative content analysis to statistical analyses as binary variables. It is not clear how this procedure influenced the results as all of the other factor variables were constructed based on structured survey questions and psychometrically validated scales. In other words, although we were able to reveal several intriguing connections between wellbeing, gameplay preferences, motives to play, and the 11 learning types, these associations could have been different in their magnitude if the data considering the learning types would have been similarly structured as the factor variables included in the analyses. Related to this issue, future research could develop the 11 learning categories into a survey inventory and triangulate the analyses of the current study by making use of more structured data on the learning types. Finally, one should recognize that while learning outcome categories were identified using an open-ended approach, the learning categories were undoubtedly influenced by the researcher’s existing conceptual understanding of learning. Because of this, future research on the learning types should not try to confirm the 11 types of learning with a confirmatory factor analysis without conducting first an extensive exploratory factor analysis with an extensive pool of possible modes of informal learning from games.

This study demonstrated that it is possible to identify distinct informal game-based learner types based on players’ self-articulated learning outcomes. Furthermore, our analysis substantiated that these learner types are distinctive from each other also in relation to gameplay motives and preferences for particular gameplay activities as well as wellbeing outcomes, thus, strengthening their profiles. While we are not able to fully confirm Crawford, 1984 claim of learning being the most fundamental motive to play games, the present study indeed illustrated the intertwined nature of gameplay, learning and personal wellbeing from various different angles. The results also showed that learning is not just a spontaneous by-product of gameplay, but rather, it is entangled in a wide-ranging manner with the motivational development. Thus, while the learning gained through non-educational gameplay activity could appear as seemingly pointless or merely entertaining, the underlying purposes of this activity may well be guided by a self-determined eudaimonic desire to grow and learn. However, further research is needed to delve into the constitution and dynamics of the motivational basis of an informal player-learner.

## Author’s note

This article is partially based on a conference paper presented at ECGBL2022.

## Data availability statement

The raw data supporting the conclusions of this article will be made available by the authors, without undue reservation.

## Ethics statement

Ethical review and approval was not required for the study on human participants in accordance with the local legislation and institutional requirements. The participants provided their written informed consent to participate in this study.

## Author contributions

JV, TV, and KT contributed to the conception and the design of the study and wrote sections of the manuscript. JV organized the data, performed, and reported the statistical analyses. TV performed and reported qualitative analyses. KT contributed to the conception of well-being in the analyses. All authors contributed to manuscript revision, and read and approved the submitted version.

## Funding

This work was funded by the Business Finland (9214/31/2019), Academy of Finland (Centre of Excellence in Game Culture Studies, decision 353267; PROFI 7 JYU.LearnDigi, decision 353325), and Kone Foundation (201908388).

## Conflict of interest

The authors declare that the research was conducted in the absence of any commercial or financial relationships that could be construed as a potential conflict of interest.

## Publisher’s note

All claims expressed in this article are solely those of the authors and do not necessarily represent those of their affiliated organizations, or those of the publisher, the editors and the reviewers. Any product that may be evaluated in this article, or claim that may be made by its manufacturer, is not guaranteed or endorsed by the publisher.
