# Supplementary material for: Informal learning and wellbeing outcomes of gameplay and their associations with gameplay motivation
Source: Front Psychol. 2023 May 30;14:1176773. doi: 10.3389/fpsyg.2023.1176773 (PMC10267973; doi:10.3389/fpsyg.2023.1176773)
Supplement: Supplementary file 1 [file Table_1.DOCX]

**Appendix 1: Exploratory Factor analysis on the 34-MAP Inventory**

**Appendix Table 1**. *The inventory items of the nine-factor Motives of the Autonomous Player (34-MAP) inventory. Reporting the primary factor loadings of each item. F1=Immersive Agency, F2=Nostalgia, F3=Social, F4=Competitive Mastery, F5=Utility, F6=Affective Engagement, F7=Escapism, F8=Addiction, F9=Boredom.*

*Why do you play videogames? Read each statement and choose the option that applies to you the best. "I play videogames.." (1=Strongly disagree, 7=Strongly agree)*

|  | F1 | F2 | F3 | F4 | F5 | F6 | F7 | F8 | F9 | Item description |
| --- | --- | --- | --- | --- | --- | --- | --- | --- | --- | --- |
| motive1 | 0.65 |  |  |  |  |  |  |  |  | ... because I can make meaningful choices in them |
| motive2 | 0.61 |  |  |  |  |  |  |  |  | ... because I can express myself in them |
| motive3 | 0.61 |  |  |  |  |  |  |  |  | ... because I can enter a fantasy world |
| motive4 | 0.69 |  |  |  |  |  |  |  |  | ... to interact with game characters |
| motive5 |  |  |  | 0.73 |  |  |  |  |  | ... to be better than my opponents |
| motive6 |  |  |  | 0.71 |  |  |  |  |  | ... to climb a competitive ladder |
| motive7 |  |  |  | 0.65 |  |  |  |  |  | ... to work hard until I master them |
| motive8 |  |  |  | 0.61 |  |  |  |  |  | ... to keep on trying to get better in them |
| motive9 |  |  | 0.77 |  |  |  |  |  |  | ... because I enjoy interacting with other players |
| motive10 |  |  | 0.74 |  |  |  |  |  |  | ... because my friends play |
| motive11 |  |  | 0.78 |  |  |  |  |  |  | ... because it brings me closer to other people |
| motive12 |  |  | 0.88 |  |  |  |  |  |  | ... to connect with others |
| motive13 |  |  |  |  | 0.79 |  |  |  |  | ... to train my brain |
| motive14 |  |  |  |  | 0.81 |  |  |  |  | ... to keep my mind sharp |
| motive15 |  |  |  |  | 0.78 |  |  |  |  | ... to enhance my memory |
| motive16 |  |  |  |  |  | 0.59 |  |  |  | ... because I am interested in them |
| motive17 |  |  |  |  |  | 0.83 |  |  |  | ... because it is fun |
| motive18 |  |  |  |  |  | 0.81 |  |  |  | ... because it is enjoyable |
| motive19 |  |  |  |  |  | 0.78 |  |  |  | ... because it is entertaining |
| motive20 |  |  |  |  |  |  |  |  | 0.75 | ... because I am bored |
| motive21 |  |  |  |  |  |  |  |  | 0.69 | ... because I have nothing else to do |
| motive22 |  |  |  |  |  |  |  |  | 0.64 | ... to pass time |
| motive23 |  |  |  |  |  |  | 0.69 |  |  | ... because it helps me disconnect from everyday routine |
| motive24 |  |  |  |  |  |  | 0.77 |  |  | ... because it distracts me from real life |
| motive25 |  |  |  |  |  |  | 0.58 |  |  | ... to take my mind off things |
| motive26 |  |  |  |  |  |  | 0.76 |  |  | ... to forget the world around me |
| motive27 |  | 0.73 |  |  |  |  |  |  |  | ... because it reminds me of good moments in my life |
| motive28 |  | 0.79 |  |  |  |  |  |  |  | ... because it feels nostalgic |
| motive29 |  | 0.76 |  |  |  |  |  |  |  | ... because I have fond memories about games |
| motive30 |  | 0.83 |  |  |  |  |  |  |  | ... because it brings up memories |
| motive31 |  |  |  |  |  |  |  | 0.82 |  | ... because I am addicted |
| motive32 |  |  |  |  |  |  |  | 0.86 |  | ... because I cannot stop playing |
| motive33 |  |  |  |  |  |  |  | 0.75 |  | ... although I have a tough time controlling my need to play |
| motive34 |  |  |  |  |  |  |  | 0.69 |  | ... because I cannot stop thinking about playing them |

**Appendix 2: Exploratory Factor analysis on the 23-WELLBEING Inventory**

**Appendix Table 2**. *The inventory items of the three-factor WELLBEING (23-WELLBEING) inventory. Reporting the primary factor loadings of each item. F1=Identity Actualization, F2=Social Connectedness, F3=Mood & Coping.

Think about what kind of experiences you get from playing videogames. For each statement, choose the option that describes you the best. "Playing videogames… (1=Completely disagree, 7=Completely agree)*

|  | **F1** | **F2** | **F3** | **Item description** |
| --- | --- | --- | --- | --- |
| wellbeing1 |  |  | 0.58 | I feel uplifted |
| wellbeing2 |  |  | 0.81 | It improves or enhances my mood |
| wellbeing3 |  |  | 0.80 | It makes me feel better |
| wellbeing4 |  |  | 0.70 | It helps me cope with stresses and everyday problems |
| wellbeing5 |  |  | 0.88 | It relieves stress and tension |
| wellbeing6 | 0.51 |  |  | It gives me a peak experience |
| wellbeing7 | 0.43 |  |  | It does help me feel more positive about life |
| wellbeing8 |  | 0.53 |  | It does give me a sense of belonging |
| wellbeing9 |  |  | 0.72 | It does give me happiness |
| wellbeing10 |  |  |  | It does enhance my quality of life |
| wellbeing11 |  | 0.48 |  | It does give me a sense of playing a valued and vital role within a community |
| wellbeing12 |  | 0.93 |  | It does make me feel connected to others |
| wellbeing13 |  | 0.90 |  | I develop friendships |
| wellbeing14 |  | 0.92 |  | I develop bonds with other people |
| wellbeing15 |  | 0.84 |  | It provides a feeling of community |
| wellbeing16 |  | 0.86 |  | It connects me to the community |
| wellbeing17 |  | 0.52 |  | I have positive feelings about other people |
| wellbeing18 | 0.72 |  |  | It does help me to think about who I am |
| wellbeing19 | 0.71 |  |  | It does push me to achieve my highest potential |
| wellbeing20 | 0.78 |  |  | It does help me learn about myself |
| wellbeing21 |  | 0.72 |  | It does give me a social group identity |
| wellbeing22 | 0.70 |  |  | I do experience self-improvement |
| wellbeing23 | 0.42 |  |  | It does allow me to be creative |
